# The origin and evolution of the ribosome

**DOI:** 10.1186/1745-6150-3-16

**Published:** 2008-04-22

**Authors:** Temple F Smith, Jung C Lee, Robin R Gutell, Hyman Hartman

**Affiliations:** 1 BioMolecular Engineering Research Center, 36 Cummington Street, Boston University, Boston, MA 02215, USA; 2 Center for Computational Biology and Bioinformatics, University of Texas, Austin, TX 78712, USA; 3 Institute for Cellular and Molecular Biology, University of Texas, Austin, TX 78712, USA; 4 Section of Integrative Biology, University of Texas, Austin, TX 78712, USA; 5 Center for Biomedical Engineering, Massachusetts Institute of Technology, Cambridge, MA 02139, USA

## Abstract

**Background:**

The origin and early evolution of the active site of the ribosome can be elucidated through an analysis of the ribosomal proteins' taxonomic block structures and their RNA interactions. Comparison between the two subunits, exploiting the detailed three-dimensional structures of the bacterial and archaeal ribosomes, is especially informative.

**Results:**

The analysis of the differences between these two sites can be summarized as follows: 1) There is no self-folding RNA segment that defines the decoding site of the small subunit; 2) there is one self-folding RNA segment encompassing the entire peptidyl transfer center of the large subunit; 3) the protein contacts with the decoding site are made by a set of universal alignable sequence blocks of the ribosomal proteins; 4) the majority of those peptides contacting the peptidyl transfer center are made by bacterial or archaeal-specific sequence blocks.

**Conclusion:**

These clear distinctions between the two subunit active sites support an earlier origin for the large subunit's peptidyl transferase center (PTC) with the decoding site of the small subunit being a later addition to the ribosome. The main implications are that a single self-folding RNA, in conjunction with a few short stabilizing peptides, formed the precursor of the modern ribosomal large subunit in association with a membrane.

**Reviewers:**

This article was reviewed by Jerzy Jurka, W. Ford Doolittle, Eugene Shaknovich, and George E. Fox (nominated by Jerzy Jurka).

## Background

The ribosome is made up of two major subunits: the large ribosomal subunit (LSU) and the small ribosomal subunit (SSU). A typical bacterial example is in *E. coli*. The small ribosomal subunit is composed of a 16S ribosomal RNA (1540 nucleotides) and 24 ribosomal proteins. The large ribosomal subunit of *E. coli *is composed of a 23S ribosomal RNA (2913 nucleotides) and 34 ribosomal proteins. The protein distribution is customarily written as (24/34) meaning 24 ribosomal proteins in the SSU and 34 ribosomal proteins in the LSU.

The bacterial ribosome has 58 proteins (24/34) as compared to the Archaea, which has 68 proteins (28/40) and the Eukarya, which has 78 proteins (32/46). There are 34 ribosomal proteins (15/19) identified as homologs in the three cellular domains [1]. When each of these universal proteins is aligned across all three cellular domains, it individually breaks up into four types of blocks: 1) universal blocks that align across the all three cellular domains; 2) blocks that align only among the Bacteria; 3) blocks that align only among the Archaea and Eukarya; and 4) domains that align only among the Eukarya [2,3].

One possible conjecture is that the universal sequence blocks of these ribosomal proteins and their interactions with the ribosomal RNAs could map out a simpler and perhaps older ribosome especially at the decoding site of the SSU and the PTC (peptidyl transferase center) of the LSU. This conjecture appears to account for the universal blocks of the ribosomal proteins of the SSU at the decoding site, but this is not the case for the ribosomal proteins of the LSU at the PTC. The observed differences between the universal blocks of the ribosomal proteins of the LSU and SSU at their active sites provide further insight into their early evolutionary history.

We assume that the different or individual biochemical processes involved in translation such as the PTC and the decoding function of the SSU arose sequentially and independently. For example, it has been suggested that an RNA molecule with a peptidyl transferase activity existed before the full sequential three-base decoding [4-6].

The structures of most tRNAs have a cloverleaf secondary structure. There are two important short helical arms, one containing the anticodon loop and the other the aminoacyl stem with its 3' CCA acceptor of the amino acid. The two other arms are terminated by the D loop and TΦ CG loop respectively. The 3D structure of the tRNA can be fitted to an L-shaped structure with the amino acid acceptor arm at one end of the L and the anticodon loop at the other. The D loop and the TΦ CG loop fold as the corner of the L. The separation between these two sites is reflected in the interaction between the tRNA and the two ribosomal subunits. The aminoacyl stem fits into the active site of the LSU while the anticodon stem fits into the decoding or active site of the SSU [7]. This raises the evolutionary question of why there are two separate subunits of the ribosome interacting with the widely separated arms of the activated tRNA. The main implications are that a single self-folding RNA, in conjunction with a few short stabilizing peptides, formed the precursor of the modern ribosomal large subunit in association with a membrane. In addition it would appear that the small subunit was a later addition.

## Results

### SSU Decoding site RNA

**Figure 1:**
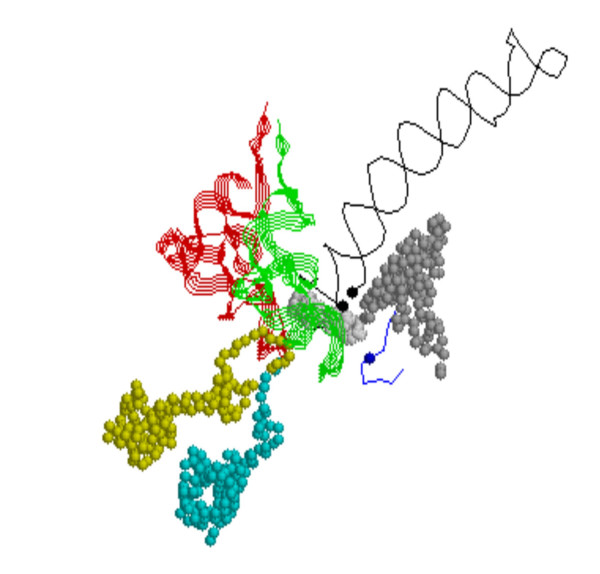
**SSU Decoding site RNAs.** Shown are the A- and P-site tRNAs as green and red strand ribbons, the mRNA fragment as space filled, and two segments of the 16S RNA in backbone (the self-folding long helix 1402–1498 (1GIX.pdb) containing the conserved A-1492 and A-1493 as black dots and the short segment containing G-530 shown as a black dot. The proteins: S9 is displayed as yellow, S12 as cyan, and S13 as gray.

There is no single self-folding segment in the 16S RNA that encompasses the majority of the decoding site rRNA. There are two segments of the 16S RNA of significant length 1402–1498 and 588–754 that are clearly capable of self-folding to their native structure in isolation [8]. The coordinates are from 1GIX.pdb [9,10]. Equivalent coordinates are in 1FJG.pdb [11]. The first of these forms a long single hairpin RNA helix whose open end points directly at the decoding site opposite the ribosomal proteins S9 and S13. This RNA helix contains, as noted by Ogle and Ramakrishnan [12], the two bases, A-1492 and A-1493, at its open end that play a key role in stabilizing the tRNA anticodon mRNA helix. This stabilization is done coordinately with the base at G-530, which is nearly a thousand nucleotides distant (Fig. 1). It is noteworthy that this large self-folding helix is truncated in minimum SSU [13]. The second RNA segment with reasonable self-folding potential, 588–754, however, is far from the decoding center. There are at least three other disjoint short segments considered part of the decoding site, 954–957, 1051–1057 and 1193–1199, largely forming one side of the tRNA pocket. None of these short segments is contained within longer segments with any self-folding potential. The lack of any continuous RNA segment of sufficient length to be capable of self-folding to the native structure of the decoding site is in strong contrast to what is observed in the LSU.

### SSU Universal Proteins

There are 15 universal ribosomal proteins associated with the SSU. These proteins can be characterized by their 3D structure, their RNA interaction and their sequence block structure [3]. These sequence blocks are either universal, with recognizable homologs within all cellular domains or unique to particular cellular domains. We restrict our study to the cellular domains of Archaea and Bacteria, due to the available 3D structures of their ribosomes and bound ribosomal proteins [9].

Five of the 15 universal SSU proteins (S2, S3, S4, S14, S15) are globular; a second group of six (S7, S9, S11, S12, S13, S19) have a globular portion plus a long unstructured peptide extension. Extensions here refer to segments of these proteins that extend away from the more compact or globular part of the protein for a significant distance. There are three ribosomal proteins (S5, S8, S10) with hairpin extensions and one (S17) with both helical and hairpin extensions. These SSU universal ribosomal proteins and their sequence blocks have functions involved with: the folding of the SSU RNA; stabilization of the folded ribosomal SSU RNA; constraining or stabilizing the tRNAs; structural interactions with other ribosomal associated proteins, e.g., initiation, elongation and termination factors, etc. and modulating the binding to the large subunit of the ribosome [9].

Hairpin extensions within the universal blocks of S10 and S5 reach toward the underside of the decoding site yet do not directly make contact. These extensions reach deep into the RNA structure probably providing RNA stabilization. Both S3 and S4 are found largely on the surface at the end of the mRNA groove, with the hairpin extension of S3 approaching the bottom of the groove but not contacting that site. The other universal SSU proteins are found largely on the SSU surface and do not appear to interact with the decoding function. S19 in the bacterial case has a short N-terminal extension that reaches toward the two tRNAs, but does not actually contact either of the tRNAs while contacting S13, which does directly interact with the decoding function. However, in the archaeal S13 case there is an additional noncellular domain-specific N-terminal block [2,3] that may reach farther but this is not available in the current determined structures.

Some distance away from the decoding site are a number of other SSU universal proteins that have a complex of cellular domain-specific blocks in both Bacteria and Archaea. These include S15, S4, S3 and probably S8. All are largely on the SSU outer surface. The primary 16S RNA contacts by these are made by the universal blocks. They also make no significant contact with the LSU. On the other hand, there is evidence that at least three, S4, S8, S15, along with S7 are critical in the early 16S protein binding and/or folding pathway [14].

### Decoding Site Proteins

The ribosomal proteins in the second group with their long extensions have the major contacts with the decoding site. Five proteins, S7, S9, S12, S13, and S11 have active site contacts to the tRNA binding site and/or the messenger RNA. Three proteins, S9, S12 and S13, contact the A or P site tRNAs (Fig. 1). The bacterial protein S9 contains only universal blocks, which are blocks or segments alignable across all Bacteria and Archaea. The contact by the S9 C-terminal extension (107–128) is primarily with the P-Site tRNA.

S13 has an irregular elongated globular domain with long C-terminal extension that makes contact between the two tRNAs at the A- and P-sites. The archaeal S13 contains significant additions or block segments not found in Bacteria. However these archaeal blocks are not involved in the S13 contacts to the tRNAs at the A- and P-sites.

The globular body of S12 on the SSU upper surface is involved with the SSU-LSU interface. A small beta hairpin (38–54) within a universal block makes contact with the decoding site and probably the mRNA. There is a very short N-terminal extension of the bacterial S12 that is unalignable with the larger N-terminal archaeal-specific block [2,3]. While the archaeal S12 long extensions probably play a role in the 16S RNA structure, they point away from the decoding site.

S7 and S11 also contact the mRNA, but at the far end of the messenger path and are largely on the surface of the SSU. S7 contacts the mRNA with the hairpin (75–87) within a universal block. S11 contains a single universal block except for a seven-amino acid deletion in the middle of the bacterial protein. The S11 mRNA contacts are made by an irregular loop within this universal block (45–58). There is an S11 long C-terminal extension that extends deep into the rRNA folded structure but away from the decoding site.

### Conserved Amino acid-Base contacts

There are a limited number of conserved amino acids contacting conserved bases among the universal proteins of Bacteria and Archaea. There are a few highly conserved amino acid positions involving Glycine, Lysine, Arginine and Aspartic acid that contact 16S conserved base positions. Among the universal proteins S9, S12 and S13 contacting the decoding site, there are very few coordinate conserved amino acid-conserved base pairs (see Table 1). Interestingly the majority involve polar side chains, suggesting that most of the 16S associated base conservation may be incidental in that the base conservation is due to RNA structure. This is also supported by the large number of amino acid side chains contacting conserved bases but that are not highly conserved. In addition much of the remaining amino acid conservation appears to involve protein-protein and internal protein structural constraints. This supports the idea that structural contacts are more highly conserved than sequence for this ancient molecular machine. One SSU counter example appears to be S12 with 17 highly conserved amino acid-based contacting pairs. Interestingly S12 also makes significant conserved amino acid-base contacts with the LSU.

**Table 1:** The conserved amino acid and conserved base contacts between the three SSU proteins (S3, S9, S12) and S13 and the 16S RNA.

16S-0515(ec.0532)-A	1	S03-156-Arg	4
16S-0515(ec.0532)-A	1	S03-159-Gly	4
16S-0515(ec.0532)-A	1	S03-161-Glu	4
16S-1354(ec.1372)-U	2	S09-069-Gly	1
16S-1327(ec.1346)-A	2	S09-107-Arg	2
16S-1328(ec.1347)-G	1	S09-107-Arg	2
16S-1326(ec.1345)-U	1	S09-120-Arg	2
16S-1327(ec.1346)-A	2	S09-120-Arg	2
16S-1330(ec.1349)-A	1	S09-120-Arg	2
16S-0885(ec.0908)-A	1	S12-021-Lys	2
16S-0886(ec.0909)-A	1	S12-021-Lys	2
16S-1468(ec.1491)-G	3	S12-046-Lys	3
16S-0890(ec.0913)-A	1	S12-047-Lys	4
16S-0505(ec.0522)-C	1	S12-049-Asn	1
16S-0510(ec.0527)-G	1	S12-049-Asn	1
16S-0511(ec.0528)-C	1	S12-049-Asn	1
16S-0512(ec.0529)-G	1	S12-049-Asn	1
16S-0501(ec.0518)-C	1	S12-050-Ser	2
16S-0502(ec.0519)-C	1	S12-050-Ser	2
16S-0512(ec.0529)-G	1	S12-050-Ser	2
16S-0503(ec.0520)-A	1	S12-052-Leu	4
16S-0504(ec.0521)-G	1	S12-053-Arg	2
16S-0505(ec.0522)-C	1	S12-053-Arg	2
16S-0506(ec.0523)-A	2	S12-053-Arg	2
16S-0503(ec.0520)-A	1	S12-054-Lys	1
16S-0504(ec.0521)-G	1	S12-054-Lys	1
16S-0505(ec.0522)-C	1	S12-069-Tyr	4
16S-0504(ec.0521)-G	1	S12-072-Gly	4
16S-0505(ec.0522)-C	1	S12-072-Gly	4
16S-0503(ec.0520)-A	1	S12-073-Glu	4
16S-0504(ec.0521)-G	1	S12-073-Glu	4
16S-0888(ec.0911)-U	1	S12-089-Arg	4
16S-0505(ec.0522)-C	1	S12-092-Asp	1
16S-0506(ec.0523)-A	2	S12-092-Asp	1
16S-0887(ec.0910)-C	1	S12-097-Arg	2
16S-0888(ec.0911)-U	1	S12-097-Arg	2
16S-1290(ec.1309)-G	1	S13-088-Arg	2
16S-1207(ec.1226)-C	1	S13-091-Arg	1
16S-1290(ec.1309)-G	1	S13-099-Arg	1

The '1' following the base or amino acid indicates conservation of 99% and '2' better than 90% across all three phylo divisions, while '3' and '4' suggest weak conservation. The 16S coordinates are for 1FJG; those in parentheses are for the *E. coli*, 1GIXstructure.

As in the SSU, there are a limited number of LSU conserved amino acids contacting conserved bases among the universal proteins (see Table 2). Most of these conserved base contacts do not directly contact the PTC. In addition the majority are again Arginines and Glycines that, however, are themselves not well-conserved. A few highly conserved amino acids (L03 Gly -208 -210 -213) appear to allow close protein RNA packing within the determined structures. The RNA conserved bases contacting these proteins are structurally conserved by base pairing, not protein side chain interactions. What is clear in the cases of the functional analog protein pairs, L10e/L16, L15e/L31 and L44e/L33, appears true in general. Again it is structure that is conserved rather than specific atomic interactions with most bases.

**Table 2:** The list of PTC-contacting proteins.

2114(ec.2073)-C	1	L02-001-Gly	5
1875(ec.1819)-A	1	L02-120-Arg	2
1855(ec.1799)-G	1	L02-141-Pro	2
1844(ec.1788)-C	1	L02-190-Arg	2
2633(ec.2598)-A	1	L02-203-Gly	2
2633(ec.2598)-A	1	L02-204-Gly	2
2634(ec.2599)-G	1	L02-205-Gly	5
2629(ec.2594)-C	1	L02-206-Arg	5
2630(ec.2595)-G	1	L02-208-His	5
2631(ec.2596)-U	2	L02-210-Gly	5
2272(ec.2239)-G	1	L02-223-Arg	5
2545(ec.2510)-U	2	L03-002-Gln	5
2547(ec.2512)-C	1	L03-005-Arg	5
2549(ec.2514)-C	2	L03-007-Arg	5
2838(ec.2821)-A	1	L03-208-Gly	1
2839(ec.2822)-C	2	L03-210-Gly	1
1733(ec.1655)-A	1	L03-213-Gly	1
2656(ec.2621)-G	2	L03-217-Arg	2
0329(ec.0322)-A	2	L04-205-Arg	2
2561(ec.2526)-C	2	L06-155-Asn	5
2567(ec.2532)-G	2	L06-158-Asp	5
2495(ec.2460)-U	1	L10e-001-Lys	-
2522(ec.2487)-G	3	L10e-007-Arg	-
1008(ec.0912)-C	1	L10e-016-Arg	-
2519(ec.2484)-C	2	L10e-061-Ser	-
2504(ec.2469)-A	1	L10e-071-Arg	-
2504(ec.2469)-A	1	L10e-071-Arg	-
1055(ec.0956)-G	1	L10e-096-Arg	-
2283(ec.2250)-G	1	L10e-113-Met	-
2282(ec.2249)-U	1	L10e-114-Arg	-
2309(ec.2275)-C	1	L10e-115-Ala	-
2310(ec.2276)-G	2	L10e-116-Ala	-
1055(ec.0956)-G	1	L10e-118-Gly	-
2502(ec.2467)-C	1	L10e-151-Arg	-
2504(ec.2469)-A	1	L10e-152-Arg	-
2501(ec.2466)-G	2	L10e-155-Asn	-
2518(ec.2483)-C	1	L10e-156-Lys	-
2597(ec.2562)-U	3	L14-034-Val	2
1295(ec.1190)-G	1	L15-014-Gly	2
0166(ec.0196)-A	1	L15-034-Gly	2
2453(ec.2415)-G	2	L15-050-Gly	2
2274(ec.2241)-A	2	L15e-077-His	-
2274(ec.2241)-A	2	L15e-081-Arg	-
2274(ec.2241)-A	2	L15e-086-Gln	-
0844(ec.0751)-A	2	L22-131-Gly	1
0840(ec.0747)-U	1	L22-132-Arg	2
2120(ec.2079)-U	2	L44e-048-Asn	-
2468(ec.2433)-A	1	L44e-050-Gly	-
2468(ec.2433)-A	1	L44e-054-Lys	-

This includes contacts suggested by others as being part of the extant center, with the 23S RNA contacting bases conserved, at least 90%. In the case of L10e, L44e and L15e the amino acid conservation is only across Archaea and Eukarya as indicated by the dash. The bacterial L16, L31 and L33 structures are not at a high enough resolution to attempt to identify equivalent contacts with such divergent sequences and structures. The notation otherwise is the same as in Table 1.

### The Large Subunit

**Figure 2:**
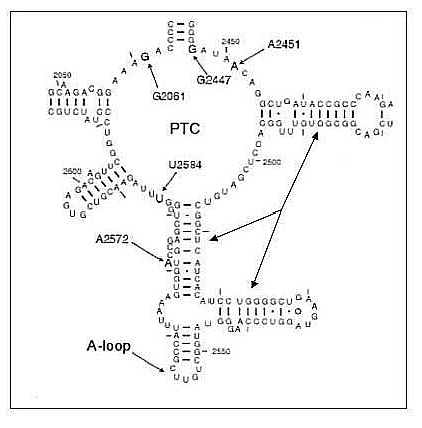
**The secondary structure of the PTC as defined by Polacek and Mankin (2005).** The equivalent base numbering is given by identifying A2451 with A2486 in reference structure 1S72.pdb. The arrows point to the helices forming the "base" of the PTC seen in Fig. 3.

A major finding from the crystal structure of the LSU demonstrated that the PTC is a ribozyme [15,16] as there are no proteins directly involved in the formation of the peptide bond. The 23S RNA segment, nucleotides 2472 through 2650, (LSU RNA coordinates are from 1S72.pdb [17,18] form the key structure of the active site, which includes the universally conserved Adenine at position 2486 adjacent to the tRNA-charged amino acid's caboxyl terminal. We calculated the free energy of this segment of rRNA in isolation and showed that the free energy was lowest when its folded secondary structure was in its native fold. This RNA segment contains two parallel helices forming the base of the A- and P-tRNA aminoacyl stem binding sites. It includes the so-called A-loop (2584–2598), which forms one side of the tRNA A-site, and a short near vertical helix (2618–2645) forming the back of the PTC. The extant PTC as defined by Bayfield *et al. *2001 [19] and Polacek & Mankin 2005 [6] includes additional bases within the segment 2091–2282 (Fig. 2). This segment forms a potentially stabilizing interaction with the segment 2623–2652 that includes the active site vertical helix. Interestingly if either of the open helical ends in Figure 2 were closed by a short loop, the entire structure would form a continuous rRNA segment of about 230 bases. There is one additional 23S RNA segment whose predicted minimum free energy secondary structure is also in its native structure. This is the segment from 2670 to 2830 forming a cruciform-like structure in the extant LSU. This cruciform structure effectively forms part of the LSU-SSU contacting surface rather than playing any role in the peptidyl transfer center, contacting the PTC base second RNA at the end opposite the site of peptidyl transfer.

**Figure 3:**
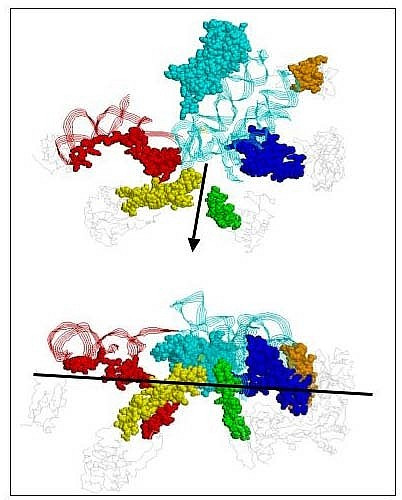
**The proposed minimal PTC.** The upper view is from above the two 23S helices that form the "base" of the PTC. The RNA segment from 2472–2650 (1S72.pdb) is shown as cyan ribbon strands representing the self-folding minimal PTC segment. The red is the adjoining small self-folding RNA additional helix 2427 to 2462, potentially displaced by L10e/L16 (see text). There are five proteins represented both in space fill and gray backbone. Their extensions are L22 (115–143) in green; L15 (1–57) in red; L6 (139–172) in orange; L4 (42–100) in yellow; L3 (1–21 and 205–262) in blue. L10e is colored cyan and is fully displayed in space fill to emphasize its mimic of a third RNA helix, forming part of the PTC base. The arrow shows the direction and location of the peptide exit tunnel. The lower figure is an on-edge view of the plane formed by the minimal PTC 23S RNA self-folding segment and the contacting peptide extensions. The black line shows the position of the plane formed by the extant PTC base RNA helices and L10e. This line also suggests a potential membrane surface upon which an early PTC function may have evolved (see Discussion section).

The L10e/L16 structural homologous pairs (Archaea and Bacteria respectively) each mimic a third RNA helix in size and shape. Their elongated globular structure is parallel with the two RNA helices forming the base of the PTC structure. The resulting three parallel "helical" bases of the PTC forms two grooves between them into which the two tRNA aminoacyl stem helices can fit [20]. This "five helical bundle" formed by the PTC plus L10e and the two tRNAs places the tRNA charged ends within a couple of angstroms of each other. This may suggest that there was an earlier third RNA helix, as part of an early self-folding PTC RNA later replaced by the proteins L10e/L16. Curiously there is a well-defined RNA helix from 2427 to 2462, such that if one calculated the minimum free energy of the longer segment 2427 through 2650 there would be a third PTC base helix available to replace the L10e/L16 helical mimic. However in the extant 23S 3D structures, the RNA helix is on the side of the LSU away from the PTC (Fig. 3).

### The LSU Proteins

There are 19 universal LSU ribosomal proteins with orthologs in the bacterial and archaeal cellular domains. Seventeen can be located on the determined 3D LSU structure [9,21]. Like the universal ribosomal proteins found on the SSU, these 17 proteins can be classified by their structures, their positions and interactions on the subunit and their cellular domain sequence block structures [3].

Their structures vary from those that are basically globular to those with or without long extensions. There are small globular proteins, L11, L23p and L29p, and those with larger globular domains, L6, L7/L12, L10e/L16 and L30. The L10e/L16 structural homolog pair has its elongated globular structure approximating the size of an RNA hairpin helix. The proteins having a globular domain with long N-terminal extensions include L2, L15, L18 and L24. L2 has both an N- and C-terminal extension.

Proteins L3, L4, L5, L13, L14 and L22 have extensions that are hairpin loops rather than N- or C terminal extensions. The L3 and L13's extensions are more complex, containing short alpha helices as well. L3's extension loop reaches deep into the rRNA structure, as does L4.

With the exception of L10e/L16 the LSU universal proteins have the majority of their globular domains on the subunit's surface. In particular L5, L11, L18, L24, L29 and L30 are nearly pure LSU surface proteins [21].

### The Proteins at the PTC

In this study we focus on the LSU proteins making significant contacts with the PTC's RNA (2472–2645). With the exception of L10e/L16 the other PTC-contacting proteins, L2, L3, L4, L6, L14 and L15e/L31 interact with the PTC through their extensions.

Of particular interest is the sequence block structure [3] of these contacting extensions. Cellular domain-specific blocks, unlike universal blocks, are defined as significant sequence segments that can be aligned as homologous only among either all Bacteria or all Archaea but not both. This distinguishes them from the universal blocks alignable across both of these cellular domains [2,3]. Many of these domain-specific sequence blocks are associated with deleted positions in the other cellular domain, while others appear to occupy similar sequence positions but have distinct amino acid composition and often differ in structural details.

The extreme case of such cellular domain specificity is found in the pairs L10e/L16, L44e/L33, L21e/L27, L15e/L31, L31e/L17, L37e/L34 and L24e/L19, which while binding nearly identical RNA substructures in Bacteria and Archaea have little or no sequence similarities and in most cases have different protein folds [21]. The pair L10e/L16, alone among these functional analog pairs, makes extensive contacts along nearly the entire length of one of the PTC base helices (2486–2533). In addition the L10e loop (97–113), which reaches the farthest along the side of the PTC (see Fig. 3), is the least similar to the bacterial L16 in structural detail. The pair L44e/L33 while not contacting the extant PTC does make extensive contacts with the RNA helix (2427 to 2462), which may have been replaced by the L10e/L16 helical mimic. The L44e/L33 protein functional analog pair also contacts the L15e/L31 functional analog pair, which also contacts that RNA helix. Does this suggest that all three of these proteins L10e/L16, L15e/L31 and L44e/L33 were added late or early as equivalent alternatives among many such [2,3]?

Both the L2 N- and C-termini contain cellular domain-specific blocks, each of which reaches in toward the top of the PTC making minimal contact. The L2 C-terminal domain-specific block is connected to the adjacent universal block by a universally conserved three-Glycine run. This would provide a unique flexible connection for this C-terminal extension. That in turn might support the idea that the two sequence blocks were once independent.

L3 contains a large complex loop (213–254) that makes contact with the PTC's second base helix. These contacts are in a short cellular domain-specific block. It also contains a unique archaeal N-terminal extension within a universal block making additional contact with the same RNA PTC helix.

L4 has a very long hairpin loop (43–100), the end of which contacts the very back of the PTC close to the universally conserved Adenine at position 2486. The end section of this L4 loop (59–83) is a cellular domain-specific block [2,3]. This L4 domain-specific block is in a nearly homologous sequence position in both Bacteria and Archaea, yet they have very different sequences and different structural details [3]. The two loops make very similar contacts to the back of PTC. In Archaea they are Ser-Gly-Arg, while in Bacteria they are Lys-Gly-Thr. Note the reversal of amino acid properties. Much of this same L4 loop contacts the L15 long N-terminal extension, which approaches the back of the PTC near the L4 PTC contact. The L15 N-terminal extension also make extensive contacts with the RNA helix, 2427 to 2462, which was potentially displaced by L10e/L16 as noted above and shown in Figure 3. The majority of the L15 N-terminal extension is within a large cellular domain-specific block [2,3].

L6 has two elongated globular domains. The contacts of L6 to the end of the first PTC base RNA helix are made by a loop connecting the last helix of one globular domain to the C-terminal strand of the other globular domain. This short loop (154–162 in Archaea) is part of an unalignable C-terminal cellular domain-specific block, unique sequence segments in both Bacteria and Archaea [2,3].

L14 has a short hairpin extension (35–45) that contacts the exterior of the PTC at the so-called A-loop RNA. This loop contains three positive amino acids and is imbedded in a universal sequence block. Interestingly all bacterial sequences to date have the actual contacting end of this loop set off by a pair of short sequence alignment deletions relative to all known Archaea. This has made it problematic to identify clearly these contacting segments as part of a universal sequence block alignable across both Bacteria and Archaea [3].

Of the above PTC contacting proteins, L3, L4, L6 and L15 appear to extend out and down from a plane formed by the PTC two-base RNA helices and the L10e/L16 helix mimic (Fig. 3). Only two proteins contacting the PTC rRNA segment are out of this plane: L2 and L14. The L14 contacts are on the exterior of the A-loop, while L2 contacts only the very top of the PTC's back forming helix. Thus the significance of these two protein's contacts for a minimal PTC function is unclear. It should be noted that the globular domains of L3 and L6 also make extensive contact with the second large 23S RNA self-folding cruciform segment (2670 to 2830). This has implications for the evolution of the ribosome, see Discussion.

### Peptide Exit Tunnel

The peptide exit tunnel, while not directly part of the PTC, is clearly an important component of the LSU's function [22] in a manner similar to the SSU mRNA groove noted above. One protein important to the tunnel is L22, whose extension (115–143) reaches deep into the LSU. This extension is within a universal block and approaches the back of the PTC similar to that of L4, while not making direct contact as does L4. The L22 extension, along with much of L4's, forms the major protein components of the exit tunnel. The extensions of L22, like those of L4 and L15, point down and out of the plane formed by the PTC base helices (Fig. 3). The globular domain of L4 and L22, along with L24 and L29, form a surface at the exit of the tunnel. The two proteins L24 and L29 make contact with the Signal Recognition Particle, SRP, complex involved with the export of new proteins into and/or through cellular membranes [23].

## Discussion

An examination of the two active sites of the LSU and the SSU shows that they differ significantly in both their RNA and protein structures and their contacts. At the RNA level the two subunit active sites are fundamentally different. The LSU PTC is composed of a contiguous self-folding segment while the SSU decoding site is composed of four to five disjoint segments, only one of which is part of a self-folding RNA segment.

At the protein level, the contacting protein extensions of these two active sites have a different character. In the SSU case, all of the contacts are within sequence segments of universal blocks alignable across all three cellular domains. While in the LSU the majority of the PTC-contacting peptide extensions are within segments of cellular domain-specific blocks. It seems characteristic of the LSU that a significant number of proteins are not only unalignable between Archaea and Bacteria but have distinct 3D structures while making nearly identical rRNA contacts. This is quite different from what is found in the ribosomal proteins of the SSU.

The above are simply observations based on our analysis, and are distinct from a couple of the more speculative implications discussed below.

### SSU Implications

Like DNA and RNA polymerases, the SSU uses a nucleic acid template and base complementarity to direct synthesis of a polymeric product. One possibility is that the first SSU was an RNA replicase that polymerized multimeric oligo nucleotides of three or more at a time. There are a number of models for how the SSU could have been an RNA replicase, all of which relate to the attachment of the anticodon loop to the codon of the messenger RNA. The triplet anticodon is then polymerized to the growing polynucleotide chain by mechanisms that are not fully specified [24-26]. Another model is based on the evolution of the tRNA from simpler precursors. This would couple the evolution of the RNA replicase to the evolution of the tRNA itself [27]. However, there are no SSU extant self-folding RNAs that could carry out this replicase function. The lack of a self-folding RNA potential ribozyme component distinguishes the active sites of the SSU from the LSU. This suggests that the precursor of the extant SSU was not evolved in the pure RNA world.

### The LSU Implications

We conclude from our study of the LSU PTC of the large ribosomal subunit that the self-folding PTC module, along with the L10e/L16 protein forms a flat surface (Fig. 3). This is the case even if the potential third PTC base helix (the red helix in Figure 3) replaces L10e. In fact an RNA segment including the two separate segments in Figure 2 if joined by one short loop also still forms a flat surface and has a reasonably high probability of self-folding. It is on the top of this RNA surface that the aminoacylated tRNA and the peptidyl tRNA can be positioned to carry out the peptidyl transfer. The structural stability and attachment to an early membrane of this PTC structure would be by the contacting peptide extensions pointing down and away from this surface. As in the case of the aminoacyl tRNA synthetases [28], micro RNA helices with an aminoacylated CCA-end could have been the original substrate of this flat RNA ribozyme and peptide complex.

From such a flat membrane surface, the large ribosomal subunit would have expanded in two directions: 1) sideways generating the exit tunnel (built from RNA and peptides of the ribosomal proteins (e.g., L4 and L22) to a site at which peptides exit (e.g., L24 and L29); and 2) eventually upwards to include the RNA segment that forms the self-folding cruciform, which is closely associated with the open end of the PTC. The upper half of the large ribosomal subunit is built from the latter structure, which interacts with the small ribosomal subunit. The simplest explanation for the evolution of the large ribosomal subunit is that it began on a membrane and gradually evolved away from this membrane into the third dimension. As the structure grew, additional peptides and proteins would have been added for stabilization and efficiency of folding a larger RNA.

### Early Membrane Implications

There are a number of options for the membrane on which the ribosome may have evolved: 1) phospho-lipid bilayer, 2) peptidyl bilayer, or 3) a mixed phospho-lipid and peptidyl bilayer. The option of a pure phosopholipid bilayer membrane raises a number of objections revolving around the problem of the impermeability of such a membrane to ions and monomers, such as nucleotides, etc. This leaves us with options 2 and 3.

The simplest hypothesis is a peptidyl bilayer as suggested by the proposed PTC anchoring peptides. Recently a number of peptides have been shown to form membranes, in particular – "The self-assembly of surfactant-like peptides containing 4–10 glycines as the component of the hydrophobic tails and aspartic acid as the hydrophilic heads is described" [29]. Thus it would appear that the LSU precursor could have evolved on a membrane totally or in part of peptides.

## Conclusion

The active site of the LSU forms a unique configuration (Fig. 3), a planar structure formed by the base RNA helices of the PTC and L10e or the potentially displaced helix 2427 to 2472 with the contacting peptides pointing down and away. This strongly suggests a potential early form of the PTC structure as a self-folding small RNA interacting with an amino acid charged RNA minihelices [30] stabilized on a membrane by a set of short peptides. The LSU's evolution of such a membrane could be viewed as a sequence of steps hinted at by its modern structure. At the decoding site of the SSU the contacting peptides are all universal cellular domain blocks. This, as discussed above, is not the case for the PTC of the LSU. The lack of a contiguous self-folding RNA forming the decoding site would imply that an early RNA polymerase SSU precursor would have been a mixture of peptides and RNAs. The peptides found at the decoding site of the SSU have not only had their structure conserved but they are alignable in sequence space across both cellular domains. This is in contrast to the peptides that interact with the PTC. These peptides share so little sequence information that they cannot be aligned while maintaining their structure. This is usually the case for very ancient proteins (e.g., tubulin and FtsZ) that retain their function via their structure rather than their sequence. This contrast between the peptide cellular domain associations of the SSU decoding site and the LSU PTC suggests that the LSU function in an early translational system preceded the SSU function. While the SSU may have begun as an RNA replicase prior to its ribosomal adaptation, that adaptation appears to have come later. It may even have come after the synthesis of larger peptides, the decedents of which are now universal SSU proteins.

## Methods

The set of tools employed included: 1) multiple sequence alignments on a wide range of representatives from Bacteria, Archaea and Eukaryota, including all those available from ribosomal subunits of determined 3D structures; 2) the generation of all contacts between the protein amino acids and rRNA bases for various inter-atomic distances; 3) association of the amino acid base contacts and the protein alignment with the cellular domain-specific sequence block structures from Vishwanath *et al*. 2004 [3] and Hartman *et al. *2005 [2]; 4) the calculation of minimum free energy predicted secondary structures for all segments of lengths between 30 and 400 nucleotides for the 16S and 23S rRNAs [8]. These were then ranked by energy, degree of helical structure and length.

These data were then examined with the aid of molecular graphics display tools using the available ribosomal 3D determined structures [17,31] with a focus on functional relationships proposed in previous published studies. In addition to the wealth of sequence and structural data, we exploited the work of Sanbonmatsu *et al. *[20] on simulating movement of tRNAs in the ribosome.

## Reviewers' comments

### Reviewer's report 1

#### Jerzy Jurka, Genetic Information Research Institute

Due to the complexity of the translation system, its origin and evolution is a notoriously difficult chicken-and-egg like problem. Most models assume that it started from a short catalytic RNA able to generate reproducible structure(s) either by self-folding or interaction with other short molecules such as peptides, or both. Following this line of thinking, the authors identified an interesting self-folding RNA segment encompassing peptidyl transfer center (PTC) in the large subunit. They speculate that this could be the original proto-ribosome. The small subunit lacks such segments near the PTC and is considered to be a later addition to the translation system. Based on the prior literature, the authors speculate that originally the small subunit might have played the role of RNA polymerase.

While the self-folding RNA segment is interesting, the discussion of protein interactions with the PTC appears to be inconclusive. On one hand, the protein contacts with the PTC appear to be conserved. However, the proteins appear to be different in Bacteria and Archaea. This leads to a rhetorical question in the middle of page 9 that should either be re-stated or removed. [Author's response: We have removed it.]

My understanding is that the data in this case do not permit to distinguish between the models of protein convergence in terms of interactions and divergence in terms of the overall structure.

[Author's response: In studying proteins such as FtsZ (bacterial structural protein) and Tubulin (eukaryotic structural protein) where there is structural but no sequential homology, it is usually assumed that there was a common ancestor and these proteins diverged from a common ancestor. We have assumed this to be true of the ribosomal proteins L10e (archaeal ribosomal protein) and L16 (bacterial ribosomal protein) and for the peptides in contact with the PTC (see paper).]

#### Other comments

It should be mentioned in the abstract that ribosomal proteins from Eukarya were not studied, otherwise sole reference to Bacteria and Archaea in the abstract is confusing.

[Author's response: See new Abstract. The eukaryotic ribosome is very similar to the archaea but there is no high resolution structure of the eukaryotic ribosome. However the domain structures of the eukaryotic ribosomal proteins have been analyzed by us (see Hartman *et al. *Archaea 2, 1–9 (2006)].

Is there any phylogenetic evidence for compensatory mutations consistent with the suggested RNA self-folding?

[Author's response: The compensatory base change has been a primary tool used to infer probable RNA secondary structure. Thus it has been known that helical regions in ribosomal RNA are identified by compensatory mutations. This is true for the PTC RNA.]

"PTC" needs to be defined when used the first time. [Author's response: PTC is defined both in the Abstract and at the first mention of it in the Background.]

Define "sequence blocks" in the text in terms of size range. [Author's response: We have added a figure at the end of these comments that gives a size distribution of the sequence blocks (details in Vishwanath *et al. *Mol. Phylogenet. Evol. 33, 615–625, 2004).]

The last column of Table 2 ranks PTC-contacting sites from 1 to 5, and some sites are hyphenated. Neither "5" nor hyphens are described. [Author's response: Figure fixed, thanks.]

Methods: it is not clear how the alignment was done. What methods/tools were used?

[Author's response: This is discussed in detail in the referenced paper, Vishwanath *et al. *Mol. Phylogenet. Evol. 33, 615–625, 2004.]

### Reviewer's report 2

#### W. Ford Doolittle, Dalhousie University

I think this is a good a useful speculation, however with more detail on ribosome structure than a non-afficionado like me can safely comment on. So all I can really address is the logic of the main conclusion, which appears on p. 13, and is succinctly summarized in the abstract. This seems a pretty reasonable scenario, though one might object that one could easily infer this without all that structural analysis: a code and a decoding site would be useless until peptides could be synthesized, after all. So what would make this paper uniquely valuable would be the novelty and strength of the arguments you mount in support of this scenario.

The weakest of these, I think, is that the greater sequence conservation of proteins involved in SSU function argues for their relative recency. Instead it might simply reflect their more intimate involvement with rRNA in maintaining the structure and function of the site.

[Author's response: It should be noted that the interaction between the SSU ribosomal proteins at the decoding site of the SSU is primarily with the phosphates in the backbone of the RNA and not with the sequences of bases in the ribosomal RNA. Thus the greater sequence conservation of the SSU ribosomal proteins is not due to the interaction with the RNA at the sequential level of nucleotides.]

And certainly one can turn such a relative conservation argument on its head: it used to be argued (by Woese and others) that those features of the translation apparatus that differ between domains do so because they arose after domain separation, which occurred while the ribosome was as yet primitive and rapidly evolving. So the structurally most conserved elements would be the oldest, functionally.

[Author's response: We agree with this concluding comment.]

### Reviewer's report 3

#### Eugene Shakhnovich, Harvard University

In this paper the authors carry out a careful and comprehensive analysis of structural and sequence alignment features of ribosomal proteins and RNA. Their principal findings are: 1) That large subunits PTC is formed by self-folding fragment of DNA and is located in locally flat area and 2) That proteins of the smaller subunit align between ribosomes from all kingdoms while proteins from LSU do not. Based on these findings the authors make some conjectures about possible evolutionary scenario by which ribosomes evolved.

The idea to infer evolutionary scenario from sequence and structural alignments is of course not novel but its comprehensive applications to ribosomes is new, to the best of my knowledge. Using ribosomes as an evolutionary case study is an excellent idea because they are presumably the most ancient molecular machines and elucidating their evolution is relevant to the key biological question of how matrix synthesis evolved.

There are some weaknesses in the argumentation. In particular the authors base their claim that LSU evolved first on the contiguity of its RNA in the PTC. However in the SSU the proteins from all three kingdoms align well while in the LSU they are not. Usually such consistent alignments is viewed as evidence that proteins belonged to LUCA – which existed before divergence form the universal common ancestor. The authors should discuss this issue.

[Author's response: The argument in this paper is based on the ribosomal proteins and their peptides at the decoding site of the SSU and contrasting them with the ribosomal proteins at the PTC site of the LSU. In fact there are a number of SSU proteins that have significant unalignable regions, for example S2, S4, and S8 (see Vishwanath *et al. *Mol. Phylogenet. Evol. 33, 615–625, 2004).]

It is not clear what is the basis of the conclusion that LSU RNA is self-folding. Is there experimental evidence, simulation or speculation? The authors should provide clear evidence in support if this central point of this study.

[Author's response: By using two different RNA secondary structure prediction computational packages, we showed that when only the PTC segment was treated as an isolated fragment its predicted structure was identical (but for one base pair) to its native whole ribosome structure. The calculated thermodynamic free energies were highly favorable for the folded structure.]

The idea that prototypical LSU was membrane bound is interesting but appears too speculative. It should be supplemented by structural and/or energetic analysis which can elucidate which membranes could LSU bind, if this hypothesis is correct

[Author's response: The membrane hypothesis is based on the interaction of the peptides at the active site of the ribosome with the PTC RNA in that they all point down and away from the flat surface formed by the self-folding PTC RNA. It should be pointed out that it is the LSU of the ribosome that interacts with a set of proteins imbedded in the membrane – a remembrance of things past.]

Overall the paper will benefit from significant revision to make it more focused on conclusions which are strongly supported by the bioinformatics analysis and which ones are more speculative.

[Author's response: We have made some modifications along these lines at the start of the Conclusion section of the paper.]

### Reviewer's report 4

#### George E. Fox, University of Houston

The manuscript entitled "The Origin and Evolution of the Ribosome" by Temple Smith and others addresses one of the single most significant questions relating to the final origin and early evolution of life. The authors build on their studies of the ribosomal proteins in which they showed that there are blocks of sequence that are universally conserved and conserved in specific Domains of life, e.g. Bacteria or Archaea. They now examine where these various blocks of conservation are in the context of key regions within the three dimensional structure of the ribosome. They combine this information with calculations of the predicted minimum free energy of large numbers of rRNA subsegments.

Using this combined approach, the authors obtain several important results. The most noteworthy being that the proposal of a minimal peptidyl transferase center that would be expected to fold correctly in the absence of other components of the modern ribosome according to thermodynamic calculations. That is of course a testable hypothesis. A second key proposal is that the PTC may have evolved in conjunction with a membrane. If correct this is an important contribution as well, as it ties together two key activities in the early history of life. The paper provides a very useful summary of protein/RNA interactions at key locations in the ribosome, e.g. PTC, exit tunnel etc. The authors argue that structure is more conserved than sequence and since structure is conserved in many interactions with the 23S rRNA without sequence conservation that is the last to go The conclusion that the core of the 50S subunit is likely older than the 30S subunit is one we have separately reached in earlier work and it is nice to see it supported here.

Overall, I strongly support publication of this manuscript in Biology Direct.

The authors may however wish to consider a variety of revisions. Most notable in this regard is the caption to Figure 3 which is very hard to relate to Figure 2. There is apparently a grammatical error in the second sentence which reads "segment from 2427–2462 (1S72.pdb) from 2472–2650 ..." makes no sentence. More to the point, it would be very helpful if one could readily relate the position numbers actually used in the text to one or more partial secondary diagrams such as Figure 2. Readability would be greatly improved if one uniform numbering system (preferably *E. coli*) were used – e.g. where is the 2427 mentioned in Figure 3 caption in Figure 2? Also, instead of continually citing structure 1S72.pdb, it would be useful to explicitly say at least once which organism it is from.

[Author's response: The Figure 2 caption has been fixed – thanks to the reviewer for noting that error. While the dual numbering does complicate the paper, the fact that the two (bacterial and archaeal) ribosome RNA structures are not alignable without numerous gaps and that there has been no agreed-upon labeling beyond that of the various RNA helices, we thought it wise to provide such independent number schemes.]

A second consideration is the apparent absence of availability of supporting materials. Table 1 summarizes a number of protein/RNA interactions. However, based on the comments in the Methods section the authors apparently have generated all such contacts for all ribosomal proteins at various inter-atomic distances. It would seem appropriate that this information and perhaps the thermodynamic calculations as well, be made available as supplementary material so others need not regenerate them.

[Author's response: Our co-authors, Robin Gutell and Jung Lee, are preparing a more expansive protein-RNA inter-atomic distance analysis for publication.]

The argument is made in the Discussion that the PTC is older than the decoding site because conserved sequence blocks in the relevant proteins are no longer universal despite the fact their structure is conserved. That is to say-structure lasts longer over evolutionary time than primary sequence. This is a solid argument that suggests that it might be useful in future work to prepare universal, Bacterial, and Archaeal "structure blocks" instead of the sequence blocks the authors have used to date.

[Author's response: A detailed correspondence between all of the identified sequence blocks with the protein three-dimensional structures is not yet complete. In the cases of those contacting the two active sites, this has been done as noted in the paper.]

Finally – a minor point; Maguire *et al. Mol Cell ***11**: 427–435 (2005) have shown that the N-terminal sequence of ribosomal protein L27 (analog is L21e in the Archaea) is very close to the active site and therefore some may argue that in Bacteria at least, that the machinery is not exclusively an RNA machine. This paper should be addressed in the subsection of the Results entitled The Large Subunit.

[Author's response: Given that this argument is only based on Bacterial L27 (N-terminal sequence) and does not appear to be the case in Archaeal L21e, we prefer to wait for further data to make the argument. The case for a peptide-RNA machine involved in the decoding site of the SSU can more easily be argued as there is no self-folding RNA that defines the site.]

## Competing interests

The authors declare that they have no competing interests.

## Authors' contributions

TFS designed the study, performed the research and helped to draft the manuscript. RG participated in the research and analyzed the data. JL participated in the research and analyzed the data. HH designed the study, performed the research and helped to draft the manuscript. All authors read and approved the final manuscript.
